# Dissociation of mitochondrial HK-II elicits mitophagy and confers cardioprotection against ischemia

**DOI:** 10.1038/s41419-019-1965-7

**Published:** 2019-09-30

**Authors:** Valerie P. Tan, Jeffrey M. Smith, Michelle Tu, Justin D. Yu, Eric Y. Ding, Shigeki Miyamoto

**Affiliations:** Department of Pharmacology, University of California, San Diego, 9500 Gilman drive, La Jolla, CA 92093-0636 USA

**Keywords:** Autophagy, Molecular biology

## Abstract

Preservation of mitochondrial integrity is critical for maintaining cellular homeostasis. Mitophagy is a mitochondria-specific type of autophagy which eliminates damaged mitochondria thereby contributing to mitochondrial quality control. Depolarization of the mitochondrial membrane potential is an established mechanism for inducing mitophagy, mediated through PINK1 stabilization and Parkin recruitment to mitochondria. Hexokinase-II (HK-II) which catalyzes the first step in glucose metabolism, also functions as a signaling molecule to regulate cell survival, and a significant fraction of cellular HK-II is associated with mitochondria (mitoHK-II). We demonstrate here that pharmacological interventions and adenoviral expression of a mitoHK-II dissociating peptide which reduce mitoHK-II levels lead to robust increases in mitochondrial Parkin and ubiquitination of mitochondrial proteins in cardiomyocytes and in a human glioblastoma cell line 1321N1, independent of mitochondrial membrane depolarization or PINK1 accumulation. MitoHK-II dissociation-induced mitophagy was demonstrated using Mito-Keima in cardiomyocytes and in 1321N1 cells. Subjecting cardiomyocytes or the in vivo heart to ischemia leads to modest dissociation of mitoHK-II. This response is potentiated by expression of the mitoHK-II dissociating peptide, which increases Parkin recruitment to mitochondria and, importantly, provides cardioprotection against ischemic stress. These results suggest that mitoHK-II dissociation is a physiologically relevant cellular event that is induced by ischemic stress, the enhancement of which protects against ischemic damage. The mechanism which underlies the effects of mitoHK-II dissociation can be attributed to the ability of Bcl2-associated athanogene 5 (BAG5), an inhibitor of Parkin, to localize to mitochondria and form a molecular complex with HK-II. Overexpression of BAG5 attenuates while knockdown of BAG5 sensitizes the effect of mitoHK-II dissociation on mitophagy. We suggest that HK-II, a glycolytic molecule, can function as a sensor for metabolic derangements at mitochondria to trigger mitophagy, and modulating the intracellular localization of HK-II could be a novel way of regulating mitophagy to prevent cell death induced by ischemic stress.

## Introduction

Mitochondria are the powerhouses that generate energy for the cell; however, when mitochondria are damaged in response to stress, they contribute to cell death^1,2^. Thus preservation of mitochondrial integrity is important for maintaining cellular homeostasis against stress. This can be achieved by inhibition of mitochondrial deterioration through activation of cell survival signaling pathways and/or by elimination of damaged mitochondria by intracellular degradation systems including autophagy and mitophagy^3,4^.

Mitophagy is a selective form of autophagy whereby damaged mitochondria are degraded in autophagolysosomes, hence protecting cells from undergoing cell death^5–8^. The means by which damaged (versus healthy) mitochondria are selected for removal is largely attributed to their specific tagging for recognition by autophagosomes. One of the most well-characterized mechanisms of tagging damaged mitochondria occurs through the loss of mitochondrial membrane potential which stabilizes PTEN-induced putative kinase 1 (PINK1) at the mitochondria and leads to Parkin recruitment. Parkin, an E3 ubiquitination ligase, then ubiquitinates outer mitochondrial membrane proteins which are subsequently recognized by autophagosomes for lysosomal degradation^9–12^. However, the physiological intracellular signaling pathways which govern this process have been largely elusive^13,14^.

Hexokinase (HK) phosphorylates glucose to produce glucose-6-phosphate, playing a critical role in glucose metabolism. Hexokinase-II (HK-II) is the predominant HK isoform in insulin-sensitive tissues including the heart, and is upregulated in many types of tumors, contributing to the Warburg effect^15^. In addition to its metabolic role, HK-II also promotes growth and increases cellular resistance to stress^16–18^. HK-II has a mitochondrial binding motif at the N-terminus and a significant portion of cellular HK-II localizes at mitochondria (mitoHK-II). MitoHK-II levels are increased in response to growth factors^19,20^ which contributes to its growth and protective effects^21,22^. On the other hand, mitoHK-II has been reported to be decreased under stress conditions^23–25^. It is not known, however, if the decrease in mitoHK-II is merely a consequence of metabolic and signaling deficiencies induced by stress or if it plays an important adaptive biological role. We hypothesized that dissociation of HK-II from mitochondria induced by ischemic stress could function as a trigger to induce mitophagy and serve as a defense mechanism.

## Materials and methods

All procedures were performed in accordance with the NIH Guide for the Care and Use of Laboratory Animals and approved by the Institutional Animal Care and Use Committee of UCSD.

### Cell culture

Neonatal rat ventricular myocytes (NRVMs) were isolated and cultured as described previously^19,20^. Briefly, myocytes were isolated from 1 to 2 day old Sprague-Dawley rats using a kit (Worthington), plated and maintained overnight in Dulbecco’s modified Eagle’s medium (DMEM) supplemented with 15% fetal bovine serum (FBS). Myocytes were starved for 24 h prior to adenoviral infection. Adenovirus for the 15NG, miniSOG-tagged PINK1, wild-type HK-II, N-terminus deletion mutant (ΔN) HK-II, BAG5 and GFP (control) were generated using Gateway Cloning Technology (Thermo Fisher Scientific). Adenovirus encoding Mito-Keima was provided by Dr. Junichi Sadoshima (Rutgers New Jersey Medical School, Newark, NJ), and adenovirus encoding mCherry-tagged Parkin (mcParkin) was provided by Dr. Asa Gustafsson (UCSD, La Jolla, CA). To knock down PINK1, Parkin or BAG5, cells were transfected with siRNA (Qiagen, 2 μg/1 × 10^6^ cells) against PINK1, Parkin or BAG5 for 48 h using DharmaFECT-1 transfection reagent. 1321N1 cells (MilliporeSigma) and clone 9 cells (ATCC) were cultured in DMEM supplemented with 10% FBS and serum starved for 24 h prior to adenoviral infection.

### Adeno-associated virus serotype 9 (AAV9) and myocardial infarction

Mice were anesthetized with isoflurane (2%) and injected via the tail vein with 80 μl of AAV9 in lactated Ringer’s solution. Myocardial infarction study was carried out by ligation of the left anterior descending (LAD) coronary artery on anesthetized mice. A left thoracotomy was performed through the third intercostal space, and under direct microscopic control, an 8-0 nylon suture was placed around the LAD coronary artery and ligated. Age-matched sham-operated control animals underwent similar surgery without ligation of the artery.

### Reagents

Primary antibodies for hexokinase-II, Parkin, COX-IV, VDAC, lamin A/C, Rho-GDI, α-actinin, cleaved-caspase-3, cleaved caspase-9, and LC-3B were from Cell Signaling Technology; PINK1 and BAG2 were from Novus Biologicals; GFP and ubiquitin were from Santa Cruz Biotechnology; and BAG5 was from Abcam. Carbonyl cyanide 4-(trifluoromethoxy)phenylhydrazone (FCCP), Bafilomycin A1 (BFA), 3-bromopyruvate (3BP), sodium iodoacetate, Evans blue and triphenyltetrazolium chloride (TTC) were purchased from MilliporeSigma. DharmaFECT 1 and LysoTracker Blue were purchased from Thermo Fisher Scientific.

### Western blot

Cells were lysed, and adult mouse ventricles were homogenized in RIPA buffer (150 mM NaCl, 50 mM Tris (pH7.4), 1% NP-40 alternative, 1% sodium deoxycholate, 0.1% SDS, 0.2 mM EDTA, supplemented with 10 μg/ml leupeptin, 10 μg/ml aprotinin, 200 μmol Na_3_VO_4_, 1 mM PMSF, and 1 mM PNPP). Samples were rocked at 4 °C for 10 min, spun down at 20,817 × *g* at 4 °C for 10 min and supernatants saved. Protein concentration was measured using micro BCA assay (Thermo Fisher Scientific). LDS sample buffer and reducing agent were added to cell lysates, and heated at 75 °C for 15 min. Equal amounts of protein (20–60 μg) were loaded onto SDS-PAGE (Thermo Fisher Scientific, NuPage system), run, and transferred to PVDF membrane (MilliporeSigma). Membranes were blocked using 5% milk in TBS-Tween at room temperature for 1 h and incubated with primary antibodies in 5% BSA/TBS-Tween at 4 °C overnight. Membranes were washed 3 × 10 min in TBS-Tween, incubated with HRP-conjugated secondary antibodies in 5% BSA/TBS-Tween at room temperature for 1 h, and washed 3 × 10 min in TBS-Tween. Membranes were developed using SuperSignal West Femto (Thermo Fisher Scientific).

### Mitochondrial isolation

Cells were washed three times with cold PBS, and harvested in mitochondria isolation buffer (420 mM mannitol, 140 mM sucrose, 2 mM EDTA, 20 mM HEPES (pH7.4), 0.025% digitonin, 10 μg/ml leupeptin, 10 μg/ml aprotinin, 200 μM Na_3_VO_4_, 1 mM PMSF and 1 mM PNPP). Cell suspensions were passed five times through a 25-gauge needle with syringe, incubated on ice for 20 min, and centrifuged at 700 × *g* at 4 °C for 10 min. Supernatants were spun at 1000 × *g* at 4 °C for 5 min again. Clarified supernatants were spun at 12,000 × *g* at 4 °C for 15 min. The supernatants were saved as the cytosolic fraction. The pellets were washed with mitochondria isolation buffer, resuspended in RIPA buffer (described above) and spun at 20,817 × *g* at 4 °C for 5 min and supernatants were saved as the mitochondrial fraction. For isolation of mitochondria from adult mouse hearts, ventricles were homogenized in mitochondrial isolation buffer and incubated on ice for 15 min. The homogenates were spun at 700 × *g* at 4 °C for 10 min, and the resultant supernatants were spun again. Clarified supernatants were spun at 12,000 × *g* at 4 °C for 15 min to yield cytosolic fractions and mitochondrial pellets. Pellets were washed with mitochondria isolation buffer, resuspended in RIPA buffer, incubated on ice for 10 min, and centrifuged at 20,817 × *g* at 4 °C for 5 min. The supernatants were saved as the mitochondrial fraction.

### Immunoprecipitation

Cells were washed with cold PBS twice and lysed in 0.3% CHAPS buffer (20 mM PIPES [pH7.2], 5 mM EDTA, 3 mM MgCl_2_, 10 mM glycerophosphate, 10 mM pyrophosphate, 0.3% CHAPS plus protease and phosphatase inhibitors). After 20 min incubation at 4 °C, samples were spun down at 20,000 × *g* for 7 min and supernatants were saved. HK-II was immunoprecipated with HK-II antibody (Santa Cruz Biotechnology) pre-coupled with Dynabeads (Dynabeads co-immunoprecipitation kit from Thermo Fisher Scientific) at 4 °C overnight. Immunocomplexes were washed with cold lysis buffer three times, eluted with elution buffer, mixed with 2X LDS and DTT, and boiled for 10 min for Western blot analysis.

### BAG5 immunofluorescence

Cells were grown on laminin coated glass coverslips in 10 cm dishes. The cells were loaded with 100 nM MitoTracker Red for 20 min, fixed for 10 min using 4% paraformaldehyde, and permeabilized using 0.1% Triton X-100 for 5 min. Cells were washed three times with TBS-Tween, blocked with 5% BSA/TBS-Tween at room temperature for 30 min, and incubated with BAG5 antibody (Abcam; diluted at 1:100 in 1% BSA in TBS-Tween) at 4 °C overnight. The cells were then washed 4 × 5 min in TBS-Tween and blocked with 5% BSA at room temperature for 10 min before addition of Alexa 488 donkey anti-rabbit secondary antibody. After 1 h of incubation with secondary antibody, cells were washed three times in TBS-Tween and mounted on glass slides using Vectorshield (Vector Labs). Pictures were acquired using a Leica SP5 confocal microscope.

### Confocal live-cell imaging

Cells were loaded with LysoTracker Blue at 250 nM for 1 h and washed twice with the modified Krebs-Henseleit buffer (121 mM NaCl, 5 mM NaHCO_3_, 10 mM Na-HEPES, 4.7 mM KCl, 1.2 mM KH_2_PO_4_, 1.2 mM MgSO_4_ and 1.8 mM CaCl_2_ and 10 mM glucose (pH = 7.4)). Mito-Keima fluorescence was visualized at room temperature using a Leica SP5 confocal microscope. Mito-Keima signal at neutral pH was excited by 458 nm laser and that at acidic pH by 561 nm, and Mito-Keima emission was detected between 610 and 640 nm. LysoTracker Blue was excited by 405 nm laser, and fluorescence was detected between 415 and 450 nm. GFP fluorescence was excited by 488 nm and detected between 500 nm and 530 nm. To measure mitochondria membrane potential, NRVMs were loaded with 50 nM of tetramethylrhodamine ethyl ester (TMRE) for 20 min at room temperature, excited at 561 nm and fluorescence was detected between 570 nm and 620 nm. Fluorescence intensity, Pearson’s coefficient, aspect ratio and form factor were measured using ImageJ2’s standard measurement tools.

### Cell death ELISA assay

Cell death was measured using the cell death detection ELISAPLUS kit (Roche Applied Science) according to the manufacturer’s instructions. Briefly NRVMs were harvested in cytosolic extraction buffer (125 mmol NaCl, 20 mmol β-glycerophosphate, 20 mmol Tris pH 7.6, 3 mmol EDTA, 3 mmol EGTA, and 0.3% P-40 alternative plus protease and phosphatase inhibitors), incubated on ice for 10 min and centrifuged at 20,000 × *g* at 4 °C for 5 min. Supernatants (10 μl) were incubated with anti-histone-biotin antibody and anti-DNA-peroxidase antibody in a streptavidin-coated 96 well plate on an orbital shaker (80 rpm) in the dark at room temperature for 2 h. Subsequently, wells were washed three times and 2,2′-azino-bis(3-ethylbenzthiazoline-6-sulfonic) acid substrate (100 μl per well) was added. Absorbance was measured at 405 nm using an Infinite M200pro plate reader (Tecan).

### Cellular ATP and hexokinase activity measurement

Cellular ATP levels were determined using the ATP determination kit (Thermo Fisher Scientific). Hexokinase activity was measured using the HK activity colorimetric assay kit (Abcam) according to the manufacturer’s instructions. Equal amounts of cell lysate (25 μg protein in 10 μl) were used for the assays. Luminescence (ATP levels) and absorbance at 450 nm (HK activity) were measured using an Infinite M200pro plate reader (Tecan).

### Statistical analysis

Results are reported as averages ± SEM. Comparison between two groups were accomplished using unpaired two-tailed Student’s test. Statistical significance was determined using ANOVA followed by the Tukey post hoc test. A *P* value of *P* < 0.05 was considered to be statistically significant.

## Results

### HK-II dissociation from mitochondria induces mitochondrial localization of exogenously expressed Parkin but not PINK1

**Fig. 1:**
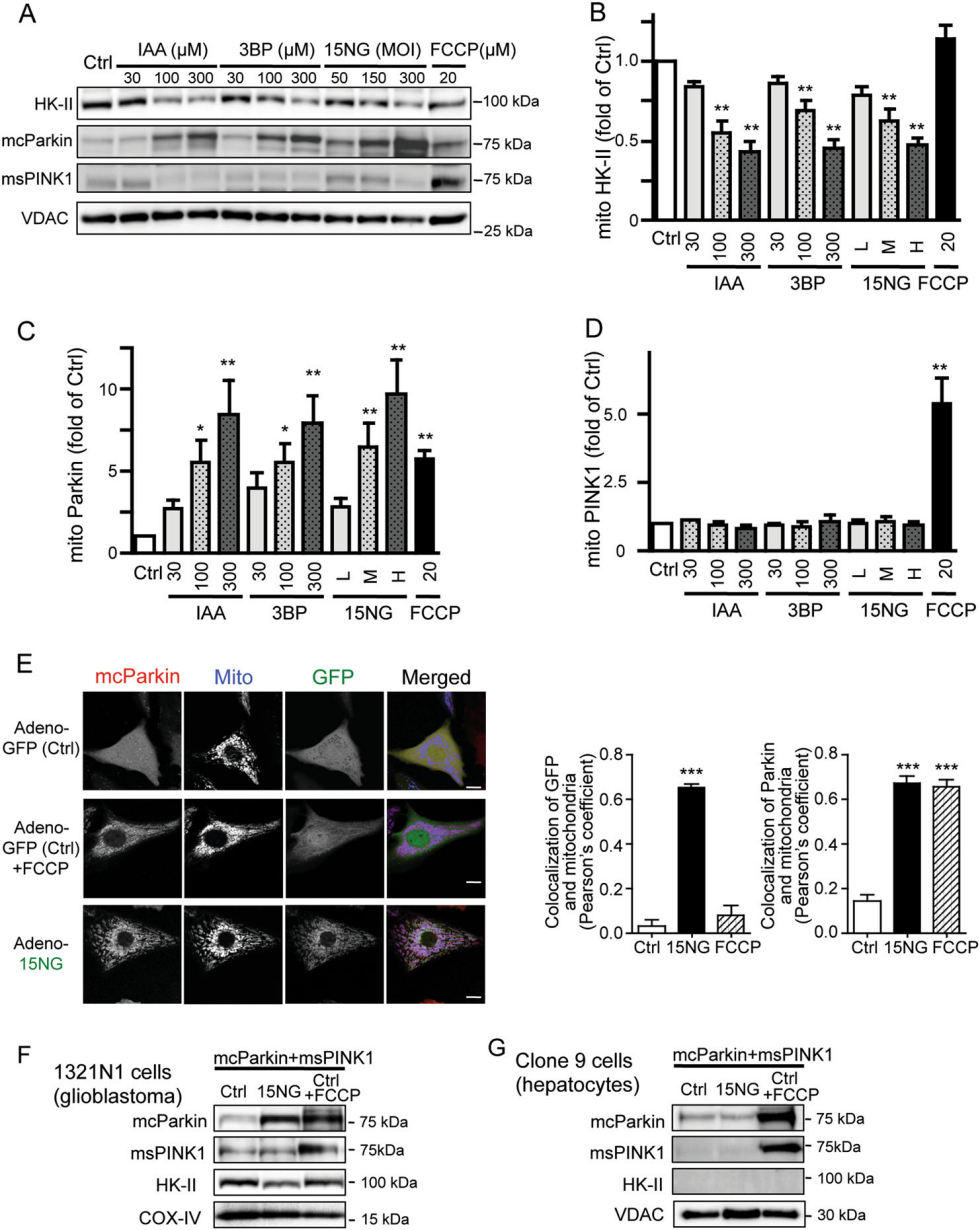
HK-II dissociation from mitochondria induces mitochondrial localization of exogenously expressed Parkin but not PINK1. **a**–**d** mCherry-tagged Parkin (mcParkin) and miniSOG-tagged PINK1 (msPINK1) were adenovirally expressed in neonatal rat ventricular myocytes (NRVMs). Cells were treated with 3-bromopyruvate (3BP), iodoacetate (IAA), or FCCP for 1 h or infected with adenovirus encoding 15NG at 50 (L), 150 (M) or 300 (H) MOI for 18 h. Mitochondrial fractions were isolated and subjected to Western blot. **b**–**d** Quantitative analysis of HK-II, mcParkin and msPINK1 levels in mitochondrial fractions. *, **p < 0.05, 0.01 versus control (Ctrl); *n* > 5. **e**. NRVMs were infected with mcParkin adenovirus and control (GFP) or 15NG adenovirus and subjected to live-cell imaging using confocal microscopy. Parkin (red); mitochondria (blue); GFP (green). Scale bars: 10 μm. Pearson’s coefficient was measured from images. *n* > 30, ***p < 0.001 versus ctrl. **f**, **g** mcParkin and msPINK1 were adenovirally expressed in 1321N1 cells or clone 9 hepatocytes. 15NG was expressed at 150 MOI. COX-IV or VDAC were used as mitochondrial markers as well as loading controls

To explore the role of mitoHK-II dissociation in regulation of mitochondrial PINK1 and Parkin levels, mCherry-tagged Parkin (mcParkin) and miniSOG-tagged PINK1 (msPINK1) were adenovirally expressed in neonatal rat ventricular myocytes (NRVMs), and mitoHK-II was dissociated using two pharmacological agents: 3-bromopyruvate (3BP)^26–28^ and iodoacetate (IAA)^24^. 3BP and IAA dose-dependently decreased the level of HK-II in mitochondrial fractions (Fig. 1a, b). The effects of 3BP and IAA are, however, not specific to inhibition of HK-II binding to mitochondria and they can be cytotoxic^29,30^. To more selectively dissociate HK-II from mitochondria, we used an adenovirus encoding a mitoHK-II dissociating peptide consisting of the N-terminus 15 amino acids of HK-II responsible for mitochondrial binding^31,32^ fused with GFP (15NG) to competitively inhibit endogenous HK-II binding to mitochondria^20^. 15NG expression at different multiplicity of infection (MOI) resulted in a dose-dependent decrease in the level of mitoHK-II (Fig. 1a, b). FCCP, a mitochondrial uncoupler, did not affect mitoHK-II levels, consistent with previous reports^24^ (also see Fig. S1). FCCP treatment induced robust increases in msPINK1 and mcParkin levels in mitochondrial fractions (Fig. 1a–d), as previously reported^9,10,12^. Dissociation of mitoHK-II by 3BP, IAA and 15NG dose-dependently increased the level of Parkin in mitochondrial fractions (Fig. 1a, c), but not PINK1 (Fig. 1a, d). Recruitment of Parkin to mitochondria was further examined by visualization of mcParkin (Fig. 1e). In control cells, Parkin (mCherry fluorescence) was uniformly distributed throughout the cell, but in cells treated with FCCP, Parkin co-localized with mitochondria. Expression of 15NG (GFP) which localized at mitochondria also led to Parkin recruitment to mitochondria, as confirmed by Pearson’s correlation coefficient analysis (Fig. 1e).

Upregulation of HK-II has been noted in cancer cells including glioblastomas^33,34^. As in cardiomyocytes, 15NG decreased HK-II in the mitochondrial fraction isolated from the 1321N1 human glioblastoma cell line (Fig. 1f) and induced an increase in mitochondrial Parkin but not PINK1 levels, while FCCP treatment increased both Parkin and PINK1. In contrast, in Clone 9 hepatocyte cells where HK-II gene expression is nearly silent^35^, 15NG expression produced no effect on either Parkin or PINK1 (Fig. 1g). These results demonstrate that the response to 15NG is dependent on HK-II expression and not restricted to cardiomyocytes.

### HK-II dissociation leads to mitochondrial localization of endogenous Parkin without inducing mitochondrial membrane depolarization and PINK1 accumulation

**Fig. 2:**
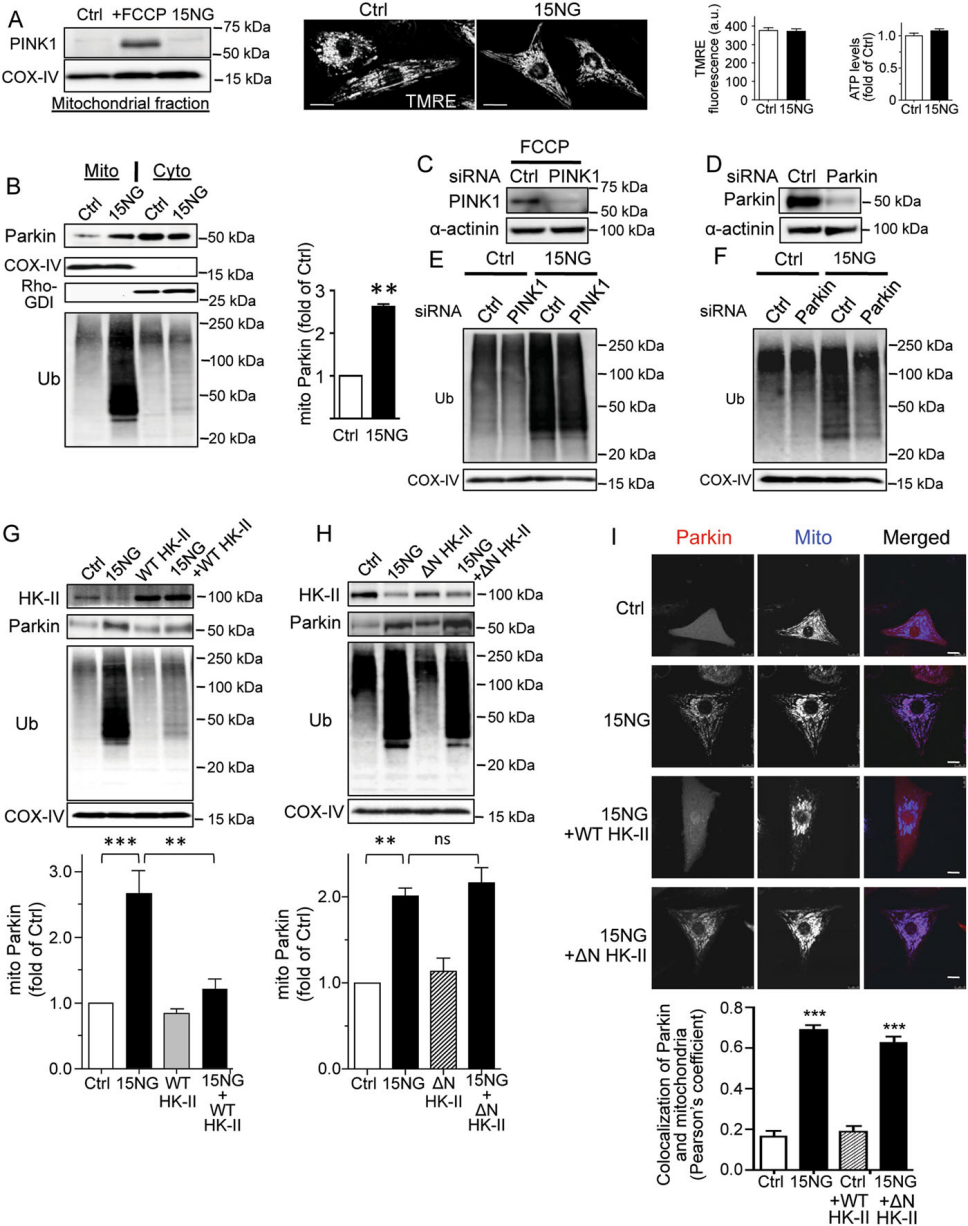
HK-II dissociation induced by 15NG expression leads to mitochondrial localization of Parkin but not PINK1 in NRVMs. **a** PINK1 Western blot was carried out on mitochondrial fractions isolated from NRVMs treated with FCCP (20 μM for 14 h) or 15NG (150 MOI for 18 h). Middle panels show TMRE fluorescence images using confocal microscopy and quantification of cellular TMRE fluorescence intensity. Scale bars: 10 μm. *n* > 60 from three independent experiments. Right panel shows cellular ATP levels in Ctrl and 15NG expressing cells. *n* = 5. **b** Mitochondrial and cytosolic fractions were subjected to Western blot for Parkin and ubiquitin (Ub). COX-IV and Rho-GDI were used as mitochondrial and cytosolic markers respectively. **p < 0.01 versus control (Ctrl); *n* = 4. **c**, **d**. Cells were transfected with siRNA against PINK1 or Parkin. PINK1 and Parkin levels were assessed by Western blot in whole cell lysates. α-actinin was used as a loading control. **e**, **f** NRVMs transfected with siRNA against PINK1 or Parkin were infected with control or 15NG adenovirus and mitochondrial fractions were subjected to Western blot. **g**, **h**. NRVMs were infected with adenovirus encoding control (GFP) or 15NG, with or without adenovirus encoding wild-type HK-II (WT HK-II, 100 MOI) or the mitochondria binding-deficient mutant HK-II (ΔN HK-II, 100 MOI). Mitochondrial fractions were subjected to Western blot. **, ***p < 0.01, *p* < 0.001; *n* = 5. **i** Parkin was visualized by mCherry fluorescence (red in the merged images) and mitochondria were visualized by MitoTracker Deep Red (blue in the merged images). Scale bars: 10 μm. Pearson’s coefficient was measured from images, *n* > 30 from four independent experiments., ***p < 0.001 versus Ctrl

The PINK1 protein undergoes constitutive degradation when mitochondrial membrane potential is intact. As expected, FCCP treatment increased the level of PINK1 in the mitochondrial fraction, whereas 15NG expression had no effect (Fig. 2a). TMRE, a mitochondrial membrane potential indicator, revealed no significant differences in mitochondrial membrane potential in control versus 15NG expressing cardiomyocytes (Fig. 2a). 15NG did not alter cellular ATP levels (Fig. 2a) or total HK activity (Fig. S2). These results suggest that mitoHK-II dissociation does not affect mitochondrial membrane potential or cellular energy status. Endogenous Parkin protein level in the mitochondrial fraction was increased by 15NG while that in the cytosolic fraction was concomitantly decreased (Fig. 2b), suggesting translocation of Parkin from cytosol to mitochondria. This was accompanied by a robust increase in ubiquitination of mitochondrial proteins (Fig. 2b). The requirement of Parkin but not PINK1 for 15NG-induced ubiquitination of mitochondrial proteins was further supported by experiments using siRNA against PINK1 and Parkin. PINK1 siRNA diminished PINK1 stabilization induced by FCCP, and Parkin siRNA decreased Parkin levels in whole cell lysates isolated from cardiomyocytes (Fig. 2c, d). PINK1 knockdown had no effect on ubiquitination of mitochondrial proteins induced by 15NG expression, whereas Parkin knockdown attenuated this response (Fig. 2e, f). To demonstrate that 15NG-induced responses are mediated by HK-II dissociation from mitochondria, mitoHK-II levels were restored by co-overexpression of wild-type (WT) HK-II. A mitochondria binding-deficient mutant of HK-II (N-terminus deletion mutant; ΔN HK-II)^20^ was used as a negative control. WT HK-II, but not ΔN HK-II, restored the level of mitoHK-II, and diminished the increases in mitochondrial Parkin and ubiquitination of mitochondrial proteins induced by 15NG (Fig. 2g, h). Co-localization of mcParkin with mitochondria was also significantly inhibited by WT HK-II but not by ΔN HK-II (Fig. 2i). These results suggest that it is HK-II dissociation from mitochondria that induces Parkin recruitment to mitochondria and ubiquitination of mitochondrial proteins in response to 15NG expression.

### MitoHK-II dissociation induces mitophagy

**Fig. 3:**
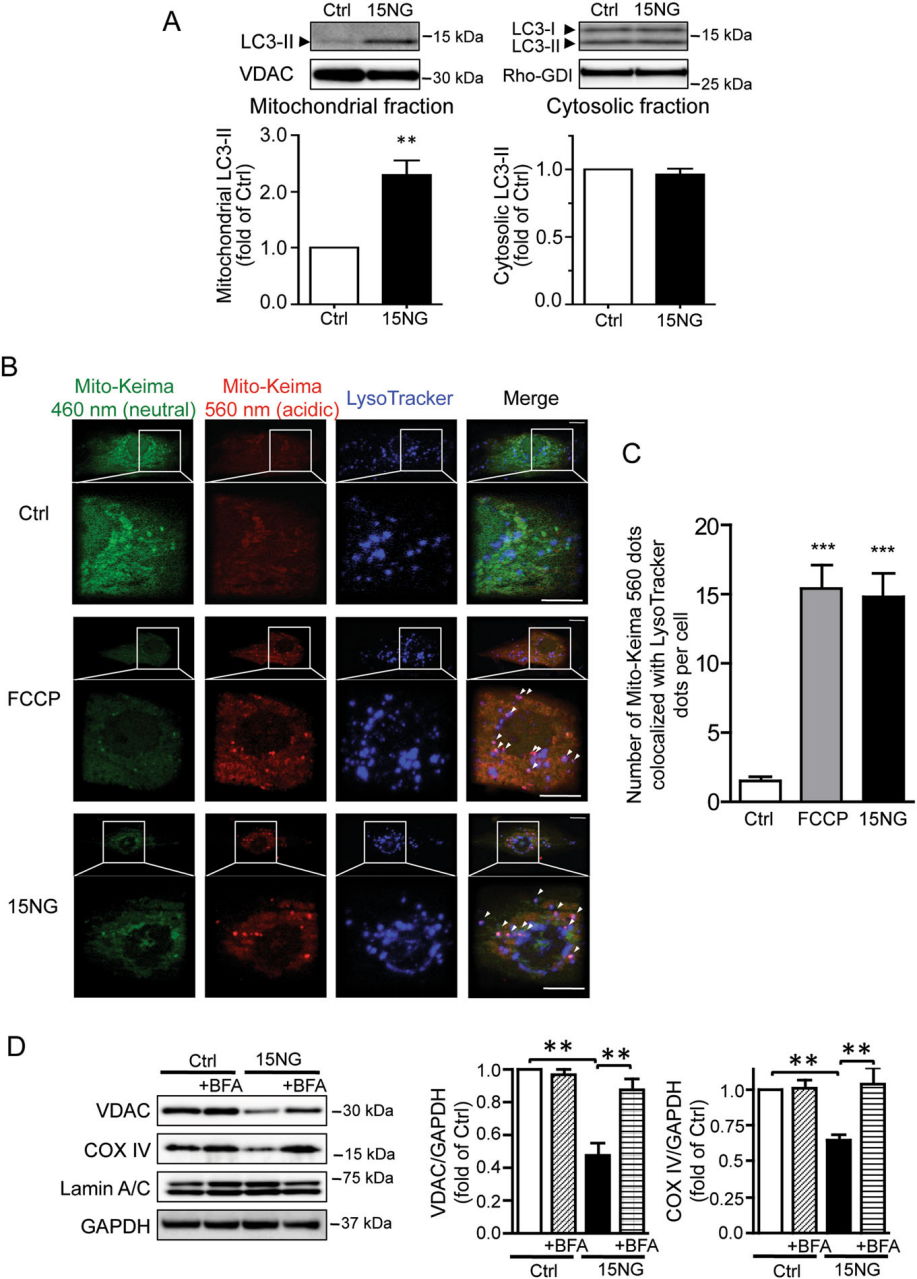
MitoHK-II dissociation induces mitophagy. **a** LC3 Western blot was carried out in mitochondrial and cytosolic fractions isolated from NRVMs expressing control (Ctrl) or 15NG (150 MOI for 18 h). **p < 0.01 versus Ctrl; *n* = 8. **b** To assess mitophagy, Mito-Keima was adenovirally co-expressed in NRVMs with Ctrl or 15NG for 32 h. LysoTracker Blue (250 nM) was loaded onto cells for 1 h prior to visualization. FCCP was used as a positive control (20 μM for 16–20 h). Scale bars: 10 μm. Arrow heads indicate the purple dots showing both MitoKeima fluorescence excited at 560 nm and LysoTracker Blue fluorescence. Data are quantified in (**c**); *n* > 40, from four independent experiments. ***p < 0.001 versus Ctrl. **d** After 48 h infection, cells were collected and subjected to Western blotting analysis of VDAC and COX IV (mitochondrial proteins), Lamin A/C (nuclear protein) and GAPDH (loading control). The lysosome inhibitor Bafilomycin A1 (BFA) was used at 50 nM. **p < 0.01; *n* = 5

LC3-II conversion from LC3-I indicates autophagosome formation. We found a marked increase in the level of LC3-II in mitochondrial fractions isolated from cells expressing 15NG (Fig. 3a), indicative of the recognition of mitochondria by autophagosomes^36,37^. In contrast, 15NG did not increase the level of LC3-II in cytosolic fractions (Fig. 3a). These results suggest that 15NG selectively induces mitophagy. To further assess mitophagy, we used mitochondrial matrix-targeted Keima (Mito-Keima), a pH-sensitive, lysosomal protease-resistant fluorescent probe^38^. Mito-Keima excitation shifts from 460 to 560 nm in the acidic environment of the lysosome. In control cells expressing Mito-Keima, we observed a fluorescence signal at 460 nm with a mitochondria-like distribution (green in Fig. 3b)^38^ while the fluorescence signal excited at 560 nm (red in Fig. 3b) was much weaker. In cells treated with FCCP, the fluorescence signal excited at 560 nm showed a distinct punctate fluorescence pattern, and many of the bright puncta co-localized with lysosomes as visualized by LysoTracker blue (purple dots indicated by arrow heads in Fig. 3b), suggesting mitochondria in the lysosome^38,39^. Remarkably, 15NG expression also increased the number of double-positive dots (Fig. 3b, c). To examine whether clearance of mitochondria is increased as a result of mitophagy induced by 15NG expression, the levels of mitochondrial proteins in whole cell lysates were examined. Following 15NG expression, VDAC and COX-IV, relative to GAPDH, were significantly decreased compared to control (Fig. 3d), and these responses were reversed by bafilomycin A1 (BFA), a lysosome inhibitor. There was no difference in the level of LaminA/C, a nuclear protein. These results suggest that in response to mitoHK-II dissociation, ubiquitinated mitochondria are recognized and engulfed by autophagosomes, delivered to lysosomes and undergo degradation.

### 15NG expression at low MOI enhances mitoHK-II dissociation and Parkin recruitment to mitochondria during ischemia and confers protection in NRVMs

**Fig. 4:**
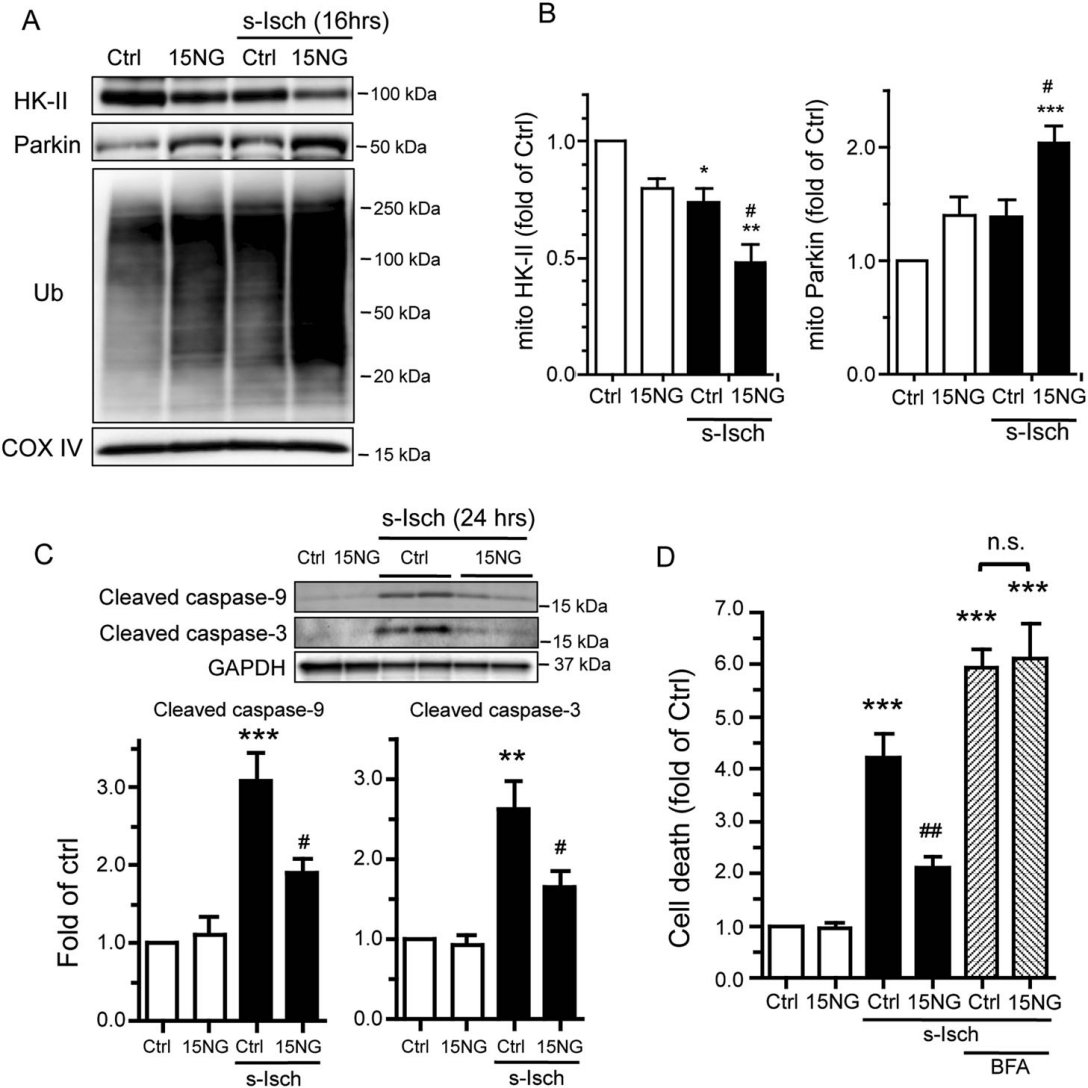
15NG expression at low MOI (50 MOI) enhances mitoHK-II dissociation and Parkin recruitment to mitochondria during ischemia and confers protection in NRVMs. **a** NRVMs were infected with control (Ctrl) or 15NG adenovirus at 50 MOI and subjected to simulated ischemia (s-Isch) for 16 h. Mitochondrial fractions were subjected to Western blot analysis for HK-II, Parkin and Ub. **b** Quantification of HK-II and Parkin levels in the mitochondrial fraction. *, **, ***p < 0.05, *p* < 0.01, *p* < 0.001 versus Ctrl. #*p* < 0.05 versus s-Isch/Ctrl; *n* = 5. **c** Western blot analysis of whole cell lysates from NRVMs for cleaved caspase-9 and cleaved caspase-3 after 24 h of s-Isch. **, ***p < 0.01, *p* < 0.001 versus Ctrl. ^#^*p* < 0.05 versus s-Isch/Ctrl; *n* = 4–5. **d** S-Isch-induced apoptosis was assessed. ***p < 0.001 versus Ctrl. ^##^*p* < 0.01 versus s-Isch/Ctrl; *n* = 4–6

We observed a reduction in mitoHK-II levels in cardiomyocytes subjected to simulated ischemia (s-Isch) as previously reported^23–25^, which was further diminished by expression of 15NG at low MOI (Fig. 4a, b). An increase in mitochondria-associated Parkin was seen with 15NG or s-Isch alone and was significantly enhanced in NRVMs exposed to the combination (Fig. 4a, b). This was accompanied by a large augmentation in ubiquitination of mitochondrial proteins (Fig. 4a). Cleaved caspase-9 and cleaved caspase-3 levels were both increased in control cells in response to simulated ischemia, but these responses were significantly attenuated in cells expressing 15NG (Fig. 4c). Further, we found that apoptotic cell death induced by simulated ischemia was considerably diminished by 15NG expression (Fig. 4d). The protective effect of 15NG was abolished by BFA (Fig. 4d), demonstrating that lysosomal degradation plays a fundamental role in 15NG-mediated protection against ischemia.

### 15NG expression in the in vivo heart confers protection

**Fig. 5:**
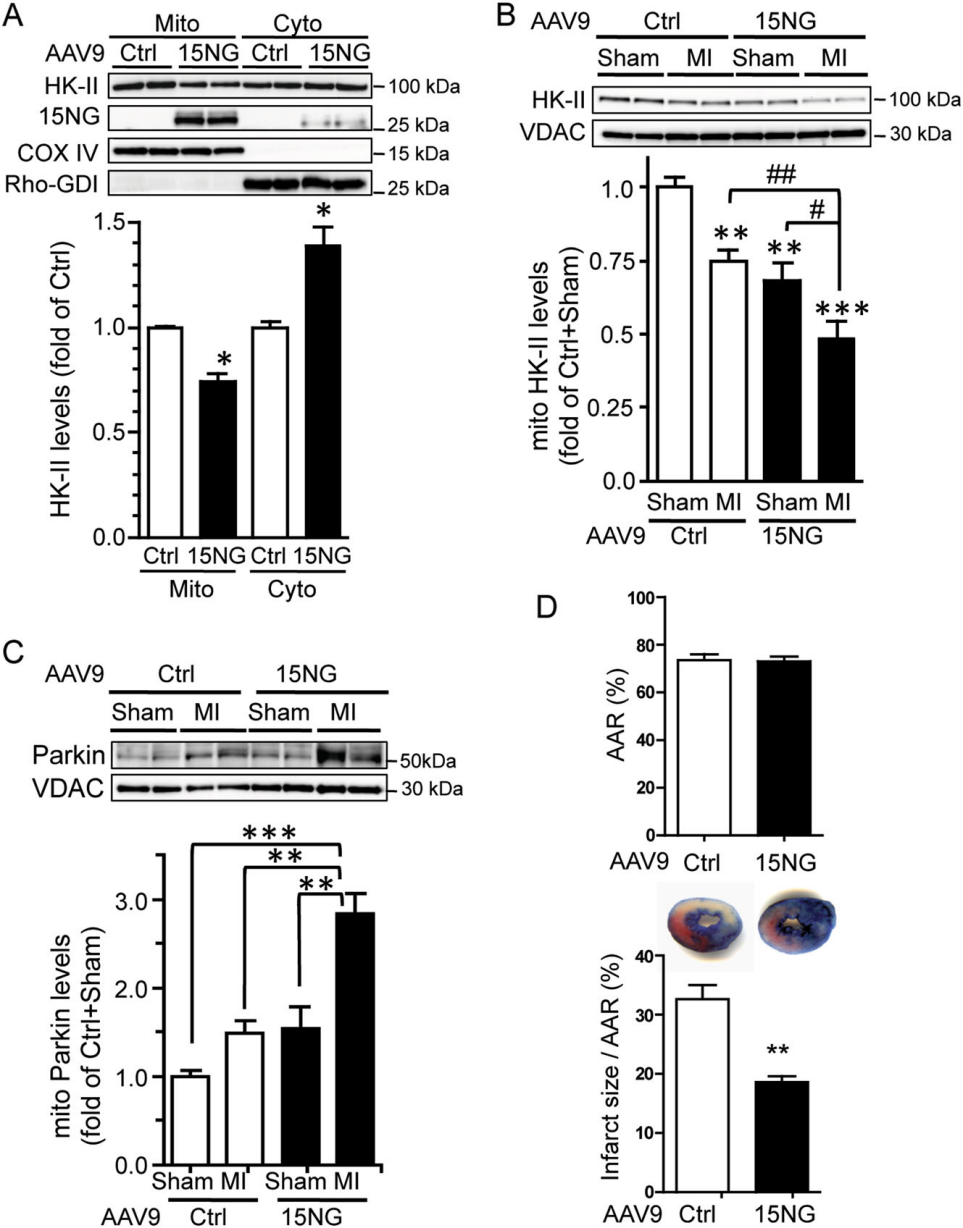
AAV9-mediated 15NG expression enhances mitoHK-II dissociation and Parkin localization to mitochondria induced by ischemia in the in vivo heart, and has a cardioprotective effect. **a** AAV9-Ctrl or AAV9-15NG was injected into mouse via the tail vein, and 2 wks later, mitochondrial and cytosolic fractions were isolated from the heart for Western blot analysis. *, **p < 0.05, *p* < 0.01 versus Ctrl; *n* = 4–5. **b**, **c** Mice were injected with AAV9-Ctrl or AAV9-15NG for 2 wks, subjected to sham surgery or regional ischemia by ligation of the left anterior descending (LAD) artery (LAD ligation; MI surgery) for 1 h, and mitochondrial fractions were isolated from the heart. **b** Western blot for HK-II in the mitochondrial fraction. **, ***p < 0.01, *p* < 0.001 versus Ctrl + Sham. ^#^, ^##^, *p* < 0.05, *p* < 0.01; *n* = 8–12. **c** Western blot for Parkin in the mitochondrial fraction. **, ***p < 0.01, *p* < 0.001; *n* = 8–12. **d** Mice injected with AAV9-Ctrl or AAV9-15NG for 2 wks were subjected to LAD ligation for 2 h, Area at Risk (AAR) and infarct size were determined by Evans blue and TTC respectively, and percentage of infarct size per AAR was calculated. **p < 0.01; *n* = 10–12

To determine the role of mitoHK-II dissociation in vivo, 15NG was expressed in the adult mouse heart using adeno-associated virus serotype 9 (AAV9, the most cardiotropic AAV serotype) driven by the cardiac-specific MLC2v promoter as previously reported^40,41^. 15NG predominantly localizes to mitochondria in the mouse heart, and its expression decreases HK-II in the mitochondrial fraction and increases it in the cytosolic fraction (Fig. 5a). The effect of 15NG expression on the response to ischemia was then examined. Mice were subjected to regional ischemia by ligation of the left anterior descending (LAD) artery (LAD ligation). MitoHK-II level was decreased in the heart subjected to LAD ligation, and was further diminished when 15NG was expressed in the heart (Fig. 5b). We did not detect changes in mitochondrial association of HK-I by ischemia (not shown), as previously reported^23,25^. Parkin levels in the mitochondrial fraction showed modest increase in response to ischemia or 15NG expression, and the increase was potentiated in mice injected with AAV9-15NG and subjected to LAD ligation (Fig. 5c), as we observed in NRVMs. To determine the effect of enhanced mitoHK-II dissociation on myocardial infarction, AAV9-injected mice were subjected to LAD ligation, and area at risk (AAR) and infarct size were determined. There was no difference in the AAR in control versus 15NG mice, but infarct size per AAR was significantly decreased in the heart expressing 15NG (Fig. 5d).

### BAG5 contributes to 15NG-induced mitophagy in NRVMs

**Fig. 6:**
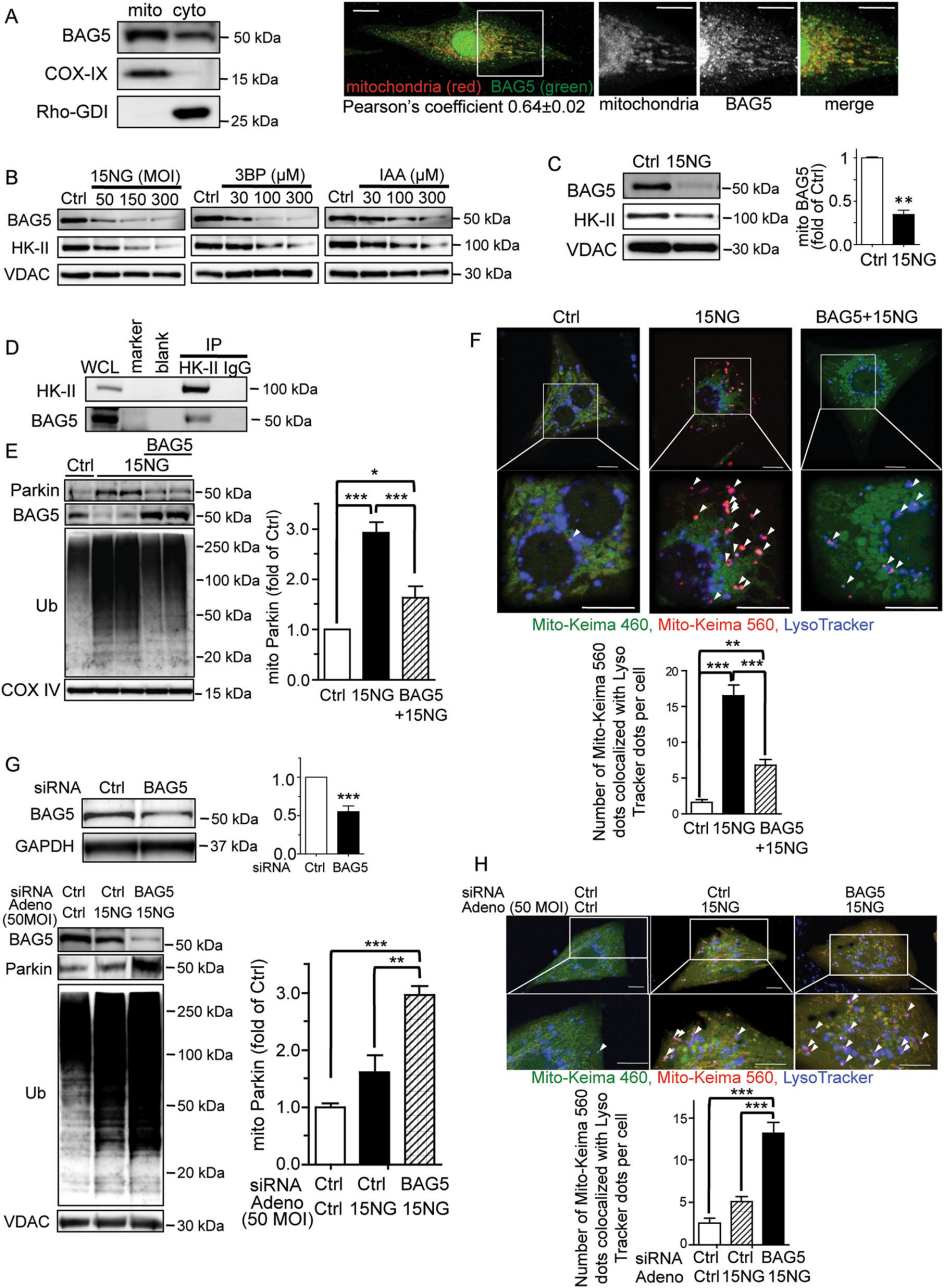
BAG5 forms an immunocomplex with HK-II and is dissociated from mitochondria by 15NG expression, contributing to 15NG-induced mitophagy in NRVMs. **a** Mitochondrial and cytosolic fractions were isolated from NRVMs for Western blot for BAG5. Immunofluorescence images show BAG5 colocalizing with mitochondria (MitoTracker Red). **b** NRVMs were subjected to various interventions to dissociate mitoHK-II (see Fig. 1 legend), and BAG5 and HK-II levels in the mitochondrial fraction were determined by Western blot. **c** Quantification of BAG5 levels in NRVMs expressing GFP (Ctrl) or 15NG. **p < 0.01 versus Ctrl; *n* = 10. **d** HK-II was immunoprecipitated from NRVMs and subjected to Western blot for BAG5. **e** BAG5 was adenovirally co-expressed in NRVMs with GFP (Ctrl) or 15NG and mitochondrial fractions were isolated and subjected to Western blot for Parkin, BAG5, Ub and VDAC. *, ***p < 0.05, *p* < 0.001; *n* = 5–7. **f** Mito-Keima assay in NRVMs expressing GFP (Ctrl) or 15NG with or without BAG5 co-expression. Arrow heads indicate double-positive fluorescence by both MitoKeima excited at 560 nm and LysoTracker Blue, seen as purple dots. Scale bars: 10 μm. The number of purple dots per cell were quantified. **, ***p < 0.01, *p* < 0.001; *n* = 50–70 from four independent experiments. **g** BAG5 was knocked-down by siRNA in NRVMs. ***p < 0.001 versus ctrl, *n* = 3. After 48 h transfection, cells were treated with Ctrl or 15NG adenovirus at low MOI (50 MOI), and mitochondrial fractions were isolated and subjected to Western blot for BAG5, Parkin, Ub and VDAC. **, ***p < 0.01, *p* < 0.001; *n* = 3-4. **h** Mito-Keima assay in NRVMs subjected to BAG5 knockdown and 15NG expression (50 MOI). Scale bars: 10 μm. ***p < 0.001, *n* > 45 from three independent experiments

Bcl2-associated athanogene 5 (BAG5), a BAG family member, has been shown to interact with and negatively regulate Parkin activity^42^. We tested the possibility that BAG5 is involved in mitoHK-II dissociation-induced Parkin recruitment to mitochondria and subsequent mitophagy. The intracellular distribution of BAG5 in cardiomyocytes was examined by Western blotting of mitochondrial and cytosolic fractions, and BAG5 was present in both (Fig. 6a). This observation was confirmed by immunofluorescence imaging, whereby a portion of intracellular BAG5 was seen to co-localize with mitochondria (Fig. 6a; Pearson’s coefficient 0.64 ± 0.02). When cardiomyocytes were treated with 15NG adenovirus, 3BP or IAA, these mitoHK-II dissociating interventions dose-dependently decreased mitochondrial BAG5 levels (Fig. 6b, c). When HK-II was immunoprecipitated from NRVMs, BAG5 was detected in the HK-II immunocomplex (Fig. 6d), indicating that HK-II and BAG5 form a molecular complex. Mitochondrial BAG5 levels decreased by 15NG expression could be recovered by adenoviral BAG5 overexpression (Fig. 6e), and remarkably, this led to a partial but significant inhibition of mitochondrial Parkin localization and ubiquitination of mitochondrial proteins induced by 15NG (Fig. 6e). Mitophagy induced by 15NG was considerably diminished by replenishment of mitochondrial BAG5 (Fig. 6f). As a converse approach, we knocked-down BAG5 by siRNA (Fig. 6g). A decrease in mitochondrial BAG5 levels induced by 15NG at low MOI was further enhanced by BAG5 knockdown. Importantly, 15NG at low MOI caused modest increases in Parkin levels in mitochondrial fractions and in the ubiquitination of mitochondrial proteins, as shown in Fig. 4, and these responses were significantly augmented by BAG5 knockdown. Remarkably, mitophagy in cells expressing 15NG was also significantly increased by BAG5 knockdown (Fig. 6h), suggesting that a decrease in BAG5 expression sensitizes cardiomyocytes to mitoHK-II dissociation-induced mitophagy.

**Fig. 7:**
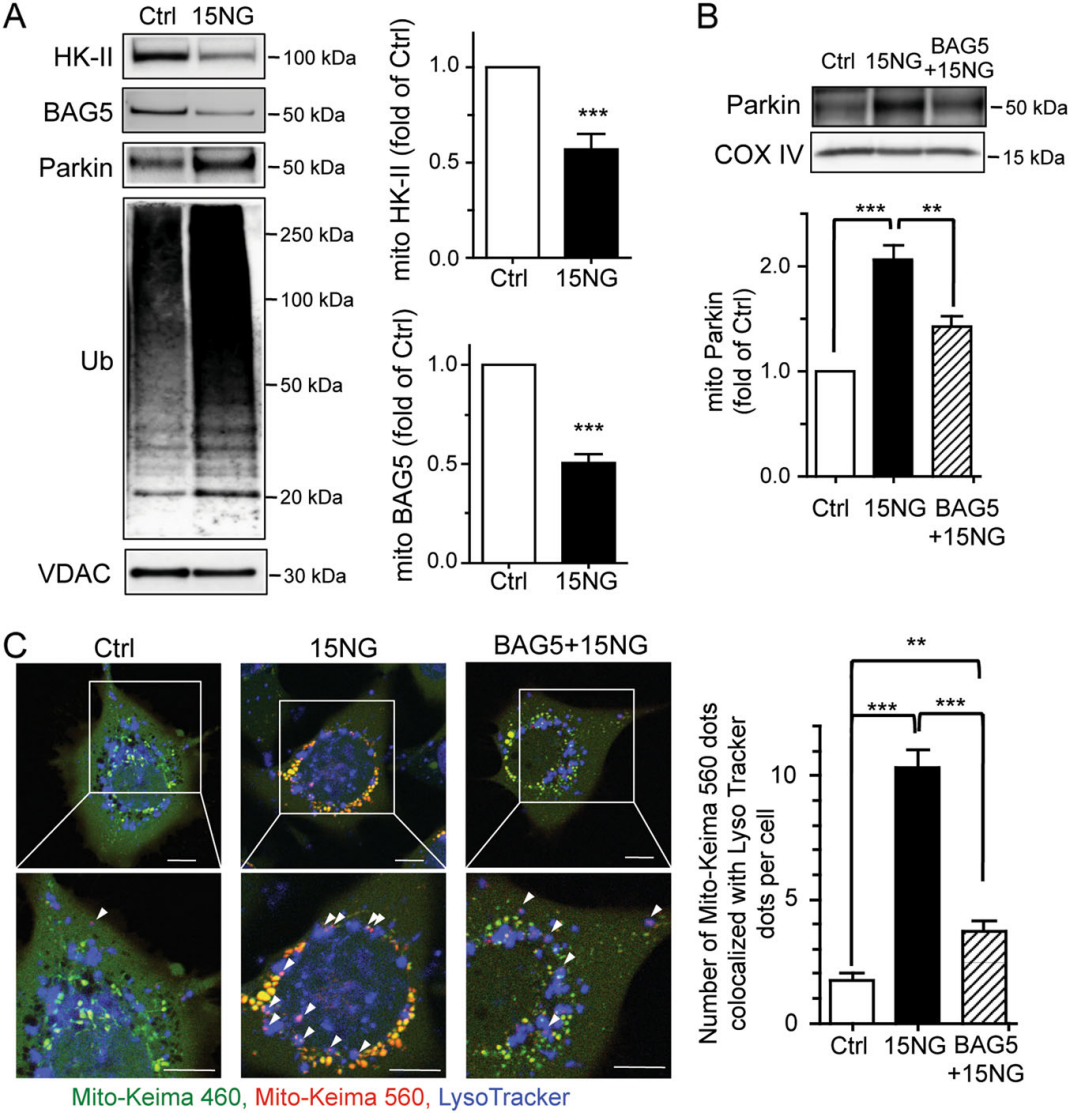
HK-II dissociation decreases mitochondrial BAG5 association and induces Parkin-mediated mitophagy in a human glioblastoma cell line 1321N1. **a** 1321N1 cells were infected with GFP (Ctrl) or 15NG adenovirus for 24 h, and mitochondrial fractions were isolated and subjected to Western blot for HK-II, BAG5, Parkin, Ub, and VDAC. ***p < 0.001 versus ctrl, *n* = 4. **b** BAG5 was adenovirally co-expressed in 1321N1 cells with GFP (Ctrl) or 15NG, and mitochondrial fractions were isolated and subjected to Western blot for Parkin and COX IV. **, ***p < 0.01, *p* < 0.001; *n* = 4–8. **c** Mito-Keima assay in 1321N1 cells expressing Ctrl or 15NG with or without BAG5 co-expression. Arrow heads indicate double-positive fluorescence by both MitoKeima excited at 560 nm and LysoTracker Blue, seen as purple dots. Scale bars: 10 μm. The number of purple dots per cell were quantified. **, ***p < 0.01, *p* < 0.001; *n* = 65–85 from four independent experiments

To determine whether HK-II dissociation from mitochondria also induces mitophagy in human cells, 15NG was expressed in 1321N1 glioblastoma cells. 15NG expression decreased the levels of mitochondrial HK-II and BAG5 and increased mitochondrial Parkin levels and the ubiquitination of mitochondrial proteins (Fig. 7a) without inducing PINK1 accumulation at mitochondria (Fig. S3). As in NRVMs, replenishment of mitochondrial BAG5 by adenoviral BAG5 overexpression diminished 15NG-mediated mitochondrial Parkin localization (Fig. 7b) and mitophagy (Fig. 7c).

## Discussion

**Fig. 8:**
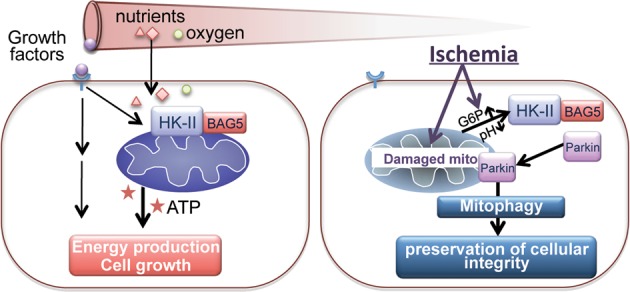
HK-II dissociation from mitochondria triggers Parkin-mediated mitophagy in response to metabolic suppression

While it is widely accepted that mitochondrial membrane depolarization promotes PINK1 stabilization resulting in Parkin recruitment to mitochondria to elicit mitophagy, many questions remain as to the exact physiological triggers of mitophagy^14^. In this study we demonstrate for the first time that dissociation of mitoHK-II induces recruitment of Parkin to mitochondria and subsequent mitophagy, and this occurs independently of mitochondrial membrane potential depolarization and PINK1 accumulation at mitochondria. Our results further suggest that the mechanism underlying the effects of mitoHK-II dissociation is related to the ability of BAG5 to localize to mitochondria and form a molecular complex with HK-II. Importantly, we demonstrate that enhanced dissociation of mitoHK-II confers protection against ischemia in vitro and in vivo. These results suggest that mitoHK-II dissociation is a signaling event that triggers protective mitophagy (Fig. 8).

### Parkin recruitment by mitoHK-II dissociation

We demonstrate that dissociation of HK-II from mitochondria induced by pharmacological interventions and adenoviral expression of a mitoHK-II dissociating peptide (15NG) induces Parkin recruitment to mitochondria. Additionally, mitoHK-II dissociation triggers ubiquitination of mitochondrial proteins and subsequent development of mitophagy in cardiomyocytes. Importantly, mitophagic effects of 15NG expression were also observed in the human glioblastoma cell line 1321N1, suggesting that mitoHK-II dissociation-induced mitophagy is not a species-specific or cell-type specific phenomenon. The ability of WT HK-II but not its mitochondrial binding-deficient counterpart (ΔN HK-II) to restore mitoHK-II levels and 15NG-induced Parkin recruitment demonstrate that the effect of 15NG is due to inhibition of HK-II binding to the mitochondria, and this is also supported by our observation that 15NG fails to induce these responses in hepatocytes in which HK-II expression is undetectable. These findings with WT and ΔN HK-II also suggest that a decrease in mitoHK-II, rather than the concomitant increase in cytosolic HK-II, is responsible for inducing Parkin recruitment to mitochondria and subsequent mitophagy. Notably, 15NG expression causes neither depolarization of the mitochondrial membrane potential, decrease in cellular ATP levels, nor PINK1 accumulation at mitochondria. Thus mitoHK-II dissociation works through a previously unidentified mechanism by which Parkin recruitment to mitochondria is regulated independent of the mitochondrial membrane potential depolarization/PINK1 pathway. Interestingly, a study using PINK1 knockout mice demonstrated that Parkin translocation to mitochondria was still evident in the heart subjected to stress^13^. This notion of PINK1-independent Parkin recruitment is supported by a recent study which showed that PINK1 was dispensable in regulating basal mitophagy in tissues of high metabolic demand including the heart^43^. Our observation that mitoHK-II dissociation by 15NG potentiates ischemia-induced translocation of Parkin to mitochondria in vitro and in vivo is in line with these findings and substantiate the existence of an alternative mechanism for activation of Parkin-mediated mitophagy.

### mitoHK-II dissociation-induced and conventional PINK1 dependent mitophagy

Our data do not rule out the possibility that mitochondrial membrane potential depolarization/PINK1 stabilization plays a role in Parkin recruitment to mitochondria under ischemia. Since 15NG does not depolarize the mitochondrial membrane potential and FCCP treatment does not decrease mitoHK-II, these two distinct pathways could converge on Parkin recruitment to mitochondria to regulate mitophagy. Indeed, we observed that ubiquitination of mitochondrial proteins induced by FCCP and 15NG was synergistic in NRVMs (Fig. S4). Mitophagy under sustained stress conditions could also be regulated in a temporal fashion by these two pathways. MitoHK-II dissociation is induced by ischemia within 1 h, while cardiac mitochondria maintain their mitochondrial membrane potential during this period of ischemia by consuming ATP^44^. Thus mitoHK-II dissociation induced by ischemia could function as the initial mitophagic event, followed by the conventional process of mitochondrial membrane depolarization-dependent PINK1 accumulation and Parkin-mediated mitophagy.

A previous study in HeLa cells showed that HK-II knockdown prevents CCCP-induced Parkin recruitment to mitochondria^45^. In cardiomyocytes, however, siRNA-mediated HK-II knockdown did not diminish FCCP-induced increases in PINK1, Parkin and ubiquitinated proteins in the mitochondrial fractions (Fig. S5). The reason for the discrepancy is not clear but could be due to differences in cell types or differences in the method of HK-II knockdown. Nonetheless, a global decrease in HK-II expression inhibits glucose metabolism through glycolysis, the pentose phosphate pathway and the hexosamine biosynthetic pathway; thus, the effect of HK-II knockdown may have a much broader ramification than that of simply manipulating its intracellular localization.

Mitochondrial fragmentation has been functionally related to mitophagy^46,47^ although its role in mitophagy is still unclear^48–50^. Recent studies suggest that balanced mitochondrial dynamics rather than morphology of mitochondria is critical in mitochondrial quality control mediated by mitophagy^51,52^. We did not observe a significant difference in mitochondrial size in cells expressing 15NG (Fig. S6). Further studies will be required to determine the role of mitochondrial dynamics in mitoHK-II dissociation-induced mitophagy.

### BAG5 involvement in mitoHK-II dissociation-induced mitophagy

In elucidating the mechanistic basis for our findings, we identified BAG5, previously reported as an inhibitor of Parkin function in neurons^42^, as a critical molecule contributing to mitophagy regulation by mitoHK-II. We demonstrate for the first time that a portion of BAG5 localizes to mitochondria and is dissociated when mitoHK-II is decreased. BAG5 dissociation from mitochondria is also observed in response to ischemia in vitro and in vivo (data not shown). We speculate that the HK-II-BAG5 complex acts as a gatekeeper at mitochondria to prevent mitophagy under basal conditions; in response to ischemic stress, its dissociation from mitochondria functions as a trigger for mitophagy. However, further studies will be required to elucidate the precise mechanism by which the mitochondrial HK-II-BAG5 complex regulates Parkin translocation and mitophagy. BAG2, another BAG family protein, has been shown to positively regulate PINK1 protein stabilization at mitochondria, thereby enhancing Parkin recruitment to mitochondria^53^. In cardiomyocytes, mitochondrial BAG2 levels were not altered by 15NG expression (Fig. S7), which is in agreement with our observation that mitoHK-II dissociation does not affect PINK1 levels at mitochondria.

### MitoHK-II dissociation and protection against ischemia

Although it is not entirely clear how ischemia decreases mitoHK-II levels, previous studies have suggested that acidosis and accumulation of glucose-6-phosphate (the catalytic product of HK), both of which are induced by ischemia^44^ (Fig. 8), negatively regulate HK-II binding to mitochondria^20,54,55^. Such direct molecular signals which arise from metabolic changes could evoke prompt protective responses against ischemia, and our results support a role for mitoHK-II as a sensor for these signals, thereby regulating mitophagy and cell survival. Interestingly, a recent study demonstrated that mitoHK-II dissociation triggers inflammasome activation in macrophages^56^, implicating a signaling role for mitoHK-II dissociation under stress conditions.

In conclusion, we present evidence for a novel mechanism for the induction of Parkin-mediated mitophagy by mitoHK-II dissociation that is independent of the canonical mitochondrial membrane potential depolarization/PINK1 pathway. Our data suggest that BAG5 contributes to mitoHK-II dissociation-induced Parkin-mediated mitophagy. Importantly, enhancing mitoHK-II dissociation during ischemia results in protection against cell death. Thus, mitoHK-II serves as a component of a cellular survival nexus: in nutrient rich conditions, it contributes to energy production^57,58^ and also resists mitochondrial death pathways in response to growth factors;^18,19,22^ on the other hand, in face of chronic ischemia, it functions as a sensor for metabolic derangements to stimulate general autophagy for energy preservation^59^ as well as signals the elimination of damaged mitochondria (Fig. 8). There is great interest in developing therapeutic interventions targeting mitophagy to treat diseases^4,6,8,60^ but the ideal targets have been elusive^60,61^. Pharmacological interventions which modulate mitoHK-II dissociation may provide a means of positively regulating mitophagy to promote cell survival during metabolic suppression.

## Supplementary information

Supplemental information
