# Supplementary material for: Dissociation of mitochondrial HK-II elicits mitophagy and confers cardioprotection against ischemia
Source: Cell Death Dis. 2019 Sep 30;10(10):730. doi: 10.1038/s41419-019-1965-7 (PMC6768853; doi:10.1038/s41419-019-1965-7)
Supplement: Supplementary file 1 — Supplemental information [file 41419_2019_1965_MOESM1_ESM.pdf]

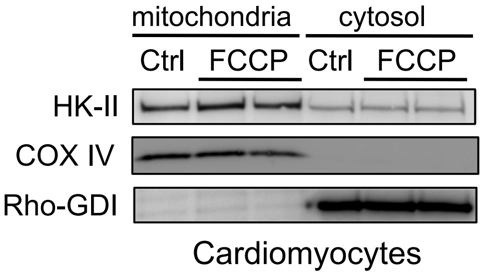

Figure S1. FCCP does not alter mitochondrial HK-II association in NRVMs. NRVMs were treated with FCCP (20 μM) and mitochondrial and cytosolic fractions were isolated for Western blot.

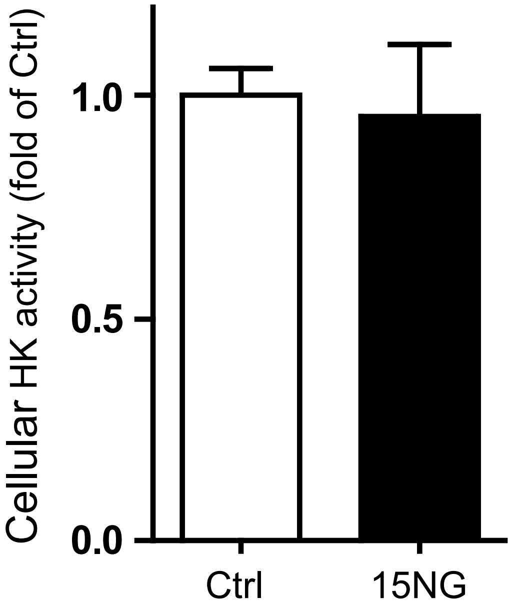

Figure S2. 15NG expression does not alter cellular hexokinase activity.
NRVMs were infected with control or 15NG and cellular hexokinase activity was measured in whole cell lysates. n=5.

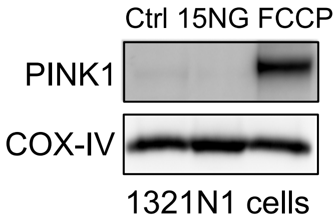

Figure S3. 15NG expression does not increase PINK1 levels in mitochondrial fractions isolated from 1321N1 cells.
1321N1 cells were infected with control or 15NG adenovirus and mitochondrial fractions were isolated for Western blot. FCCP was used as a positive control.

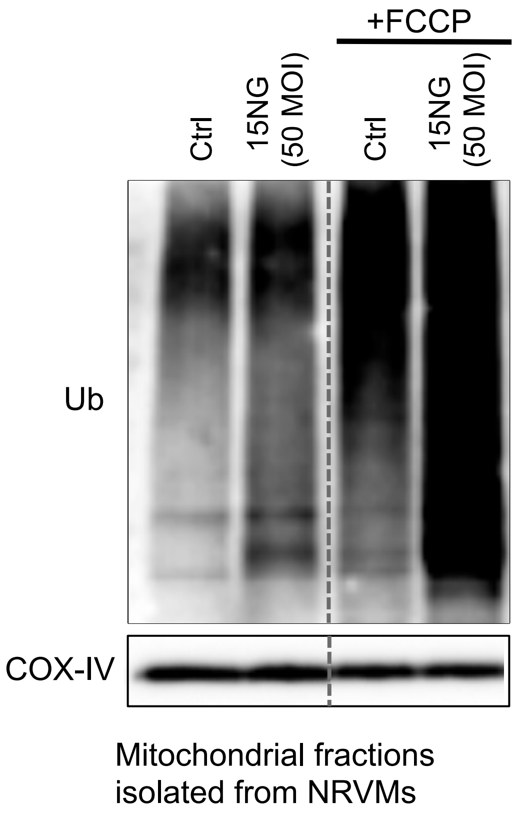

Figure S4. Ubiquitination of mitochondrial proteins induced by 15NG (50 MOI) and FCCP was synergistic in NRVMs.
NRVMs were infected with control or 15NG at low MOI (50), treated with FCCP (20 μM for 16 hrs) and mitochondrial fractions isolated for Western blot.

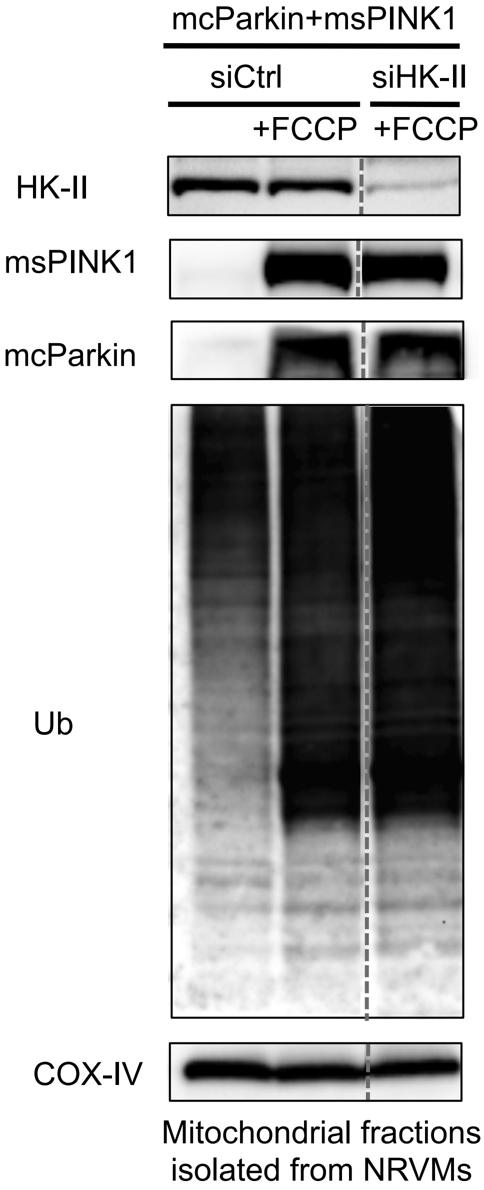

Figure S5. Knockdown of HK-II does not inhibit FCCP-induced mitophagy in NRVMs. NRVMs were transfected with siRNA-control or siRNA-HK-II for 48 hrs, infected with msPINK1 and mcParkin adenoviruses for 16 hrs, treated with FCCP and mitochondrial fractions isolated for Western blot.

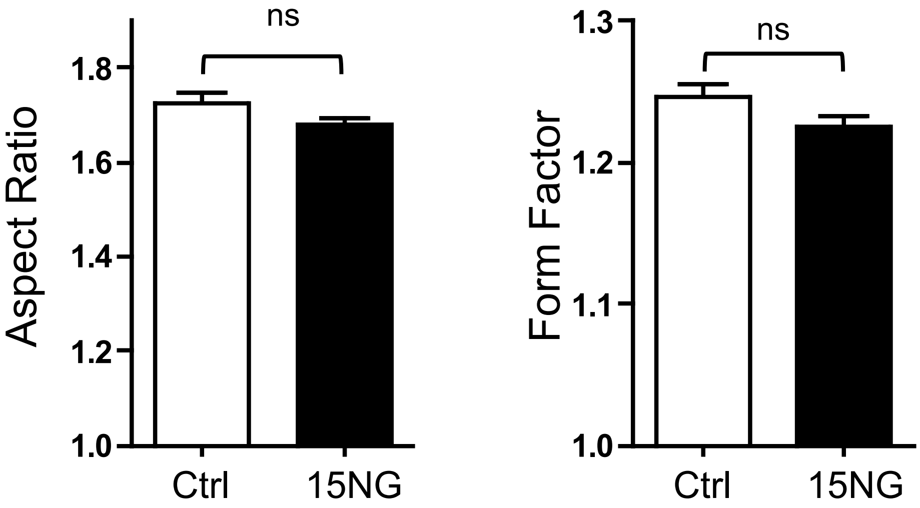

Figure S6. 15NG expression does not induce mitochondrial fragmentation. NRVMs were infected with control or 15NG adenovirus and mitochondria were visualized using MitoTracker DeepRed. Aspect ratio and form factor were analyzed using ImageJ2. ns; not significant. n>30 from 3 independent experiments.

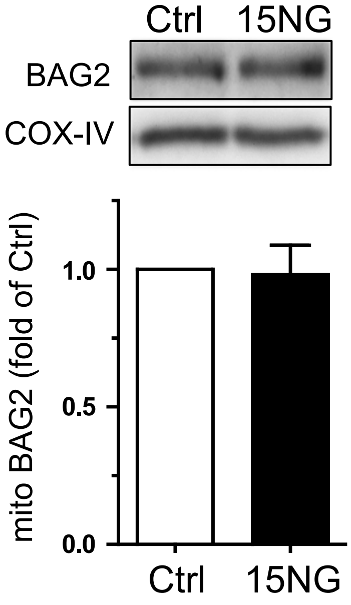

Figure S7. 15NG expression does not affect BAG2 levels in mitochondrial fractions.
NRVMs were infected with control or 15NG adenovirus and mitochondria were isolated for Western blot. n=3.
